# Supplementary material for: One-way dependent clusters and stability of cluster synchronization in directed networks
Source: Nat Commun. 2021 Jul 1;12:4073. doi: 10.1038/s41467-021-24363-7 (PMC8249607; doi:10.1038/s41467-021-24363-7)
Supplement: Supplementary file 1 — Supplementary information [file 41467_2021_24363_MOESM1_ESM.pdf]

Supplementary information for One-way dependent clusters and stability of cluster synchronization in directed networks

Matteo Lodi,[1] Francesco Sorrentino,[2] Marco Storace [1*]

[1]DITEN, University of Genoa, Via Opera Pia 11a, I-16145, Genova, Italy
[2]Mechanical Engineering Department, University of New Mexico, Albuquerque, NM 87131, USA

*To whom correspondence should be addressed; E-mail: marco.storace@unige.it.

June 16, 2021

1 Multilayer structure of a network

Following [1], in order to better evidence the layers, and the role of intralayer and interlayer connections, the network nodes can be arranged in sets $\{\mathcal{V}^\alpha, \alpha = 1, \dots, M\}$, where each set (containing N^α nodes) corresponds to a given layer of the multilayer network. The intralayer interactions inside layer α are described by an adjacency matrix $\hat{A}^{k,\alpha\alpha}$, a nonlinear coupling function $\boldsymbol{h}^{k,\alpha\alpha}$, and a coupling strength $\sigma_k^{\alpha\alpha}$. The interlayer interactions from layer β to layer α are described by an $N^\alpha \times N^\beta$ adjacency matrix $\hat{A}^{k,\alpha\beta}$, a nonlinear coupling function $\boldsymbol{h}^{k,\alpha\beta}$, and a coupling strength $\sigma_k^{\alpha\beta}$.

We now call $\boldsymbol{x}_i^\alpha$ the state of node i inside layer α ($i = 1, \dots, N^\alpha$ and $\alpha = 1, \dots, M$) and $\boldsymbol{f}^\alpha$ the dynamics of all uncoupled nodes in layer α. Then Eq. (1) in the main paper for node i in layer α can be rewritten as follows,

$$\begin{aligned}\dot{\boldsymbol{x}}_i^\alpha = \boldsymbol{f}^\alpha(\boldsymbol{x}_i^\alpha(t)) + & \underbrace{\sum_{k=1}^{L} \sigma_k^{\alpha\alpha} \sum_{j=1}^{N^\alpha} \hat{A}_{ij}^{k,\alpha\alpha} \boldsymbol{h}^{k,\alpha\alpha}(\boldsymbol{x}_i^\alpha(t), \boldsymbol{x}_j^\alpha(t-\delta_k)) +}_{\text{intralayer connections}} \\ & + \underbrace{\sum_{k=1}^{L} \sum_{\beta \neq \alpha} \sigma_k^{\alpha\beta} \sum_{j=1}^{N^\beta} \hat{A}_{ij}^{k,\alpha\beta} \boldsymbol{h}^{k,\alpha\beta}(\boldsymbol{x}_i^\alpha(t), \boldsymbol{x}_j^\alpha(t-\delta_k))}_{\text{interlayer connections}} .\end{aligned}$$

The matrix $\hat{A}^k$ is now the so-called supra-adjacency matrix for all the connections of type k of the multilayer network,

$$\hat{A}^k = \begin{bmatrix} \hat{A}^{k,11} & \hat{A}^{k,12} & \dots & \hat{A}^{k,1M} \\ \hat{A}^{k,21} & \hat{A}^{k,22} & \dots & \hat{A}^{k,2M} \\ \vdots & \dots & \ddots & \vdots \\ \hat{A}^{k,M1} & \hat{A}^{k,M2} & \dots & \hat{A}^{k,MM} \end{bmatrix},$$

which is different from the matrix A^k of Eq. (1) in the main paper.

For example, for the network in Fig. 1 of the main paper, the matrices A^k are

$A^1 =$

	①	②	③	④	⑤	⑥	⑦	⑧	⑨	⑩	⑪	⑫
①	0	1	0	1	0	0	0	0	0	0	0	0
②	1	0	0	1	0	0	0	0	0	0	0	0
③	1	0	0	1	0	0	0	0	0	0	0	0
④	1	1	1	0	0	0	0	1	0	0	0	0
⑤	0	2	1	0	0	0	0	1	0	0	0	0
⑥	0	2	1	0	0	0	0	1	0	0	0	0
⑦	0	0	0	0	1	0	0	0	0	0	0	0
⑧	0	0	0	0	1	0	0	0	0	0	0	0
⑨	0	0	0	0	0	0	0	0	0	0	0	0
⑩	0	0	0	0	0	0	0	0	0	0	0	0
⑪	0	0	0	0	0	0	0	1	1	0	0	2
⑫	0	0	0	0	0	0	0	1	1	0	2	0

and

$A^2 =$

	①	②	③	④	⑤	⑥	⑦	⑧	⑨	⑩	⑪	⑫
①	0	0	0	0	0	0	0	0	0	0	0	0
②	0	0	0	0	0	0	0	0	0	0	0	0
③	0	0	0	0	0	0	0	0	0	0	0	0
④	0	0	0	0	0	0	0	0	0	0	0	0
⑤	0	0	0	0	0	0	0	0	0	0	0	0
⑥	0	0	0	0	0	0	0	0	0	0	0	0
⑦	0	0	0	0	0	0	0	1	0	1	0	0
⑧	0	0	0	0	0	0	1	0	1	0	0	0
⑨	0	0	0	0	0	0	0	1	0	1	0	0
⑩	0	0	0	0	0	0	1	0	1	0	0	0
⑪	0	0	0	0	0	0	0	0	0	0	0	0
⑫	0	0	0	0	0	0	0	0	0	0	0	0

,

where the circled numbers indicate the nodes.

For the same network, the matrices $\hat{A}^k$ are

$\hat{A}^1 =$

$\mathcal{V}_1$			$\mathcal{V}_2$							$\mathcal{V}_3$			
①	②	③	④	⑤	⑥	⑦	⑧	⑪	⑫	⑨	⑩		
0	1	0	1	0	0	0	0	0	0	0	0	①	$\mathcal{V}_1$
1	0	0	1	0	0	0	0	0	0	0	0	②	
1	0	0	1	0	0	0	0	0	0	0	0	③	
1	1	1	0	0	0	0	1	0	0	0	0	④	$\mathcal{V}_2$
0	2	1	0	0	0	0	1	0	0	0	0	⑤	
0	2	1	0	0	0	0	1	0	0	0	0	⑥	
0	0	0	0	1	0	0	0	0	0	0	0	⑦	
0	0	0	0	1	0	0	0	0	0	0	0	⑧	
0	0	0	0	0	0	0	1	0	2	1	0	⑪	
0	0	0	0	0	0	0	1	2	0	1	0	⑫	
0	0	0	0	0	0	0	0	0	0	0	0	⑨	$\mathcal{V}_3$
0	0	0	0	0	0	0	0	0	0	0	0	⑩	

and

$\hat{A}^2 =$

$\mathcal{V}_1$			$\mathcal{V}_2$							$\mathcal{V}_3$			
①	②	③	④	⑤	⑥	⑦	⑧	⑪	⑫	⑨	⑩		
0	0	0	0	0	0	0	0	0	0	0	0	①	$\mathcal{V}_1$
0	0	0	0	0	0	0	0	0	0	0	0	②	
0	0	0	0	0	0	0	0	0	0	0	0	③	
0	0	0	0	0	0	0	0	0	0	0	0	④	$\mathcal{V}_2$
0	0	0	0	0	0	0	0	0	0	0	0	⑤	
0	0	0	0	0	0	0	0	0	0	0	0	⑥	
0	0	0	0	0	0	0	1	0	0	0	1	⑦	
0	0	0	0	0	0	1	0	0	0	1	0	⑧	
0	0	0	0	0	0	0	0	0	0	0	0	⑪	
0	0	0	0	0	0	0	0	0	0	0	0	⑫	
0	0	0	0	0	0	0	1	0	0	0	1	⑨	$\mathcal{V}_3$
0	0	0	0	0	0	1	0	0	0	1	0	⑩	

,

where the double lines separate the submatrices $\hat{A}^{k,\alpha\beta}$.

2 Adjacency matrix of the network of Fig. 2 of the main paper

$$A^1 = \begin{bmatrix} 0 & 1 & 0 & 0 & 0 \\ 1 & 0 & 0 & 0 & 0 \\ 1 & 0 & 0 & 1 & 1 \\ 0 & 1 & 1 & 0 & 1 \\ 1 & 0 & 1 & 1 & 0 \end{bmatrix}$$

3 Matrices T and B for some of the considered examples

As stated in the main paper, the structure of $B_\perp$ allows one to easily detect the presence of intertwined or one-way dependent clusters by inspection.

The complete matrices T and B, whose structure is described in this section, are provided in datasets S1 and S2.

3.1 Violin players network in its undirected configuration (high δ)

We focus on the network shown in Fig. 6 of the main paper, with the non-minimal balanced coloring shown in panel c. Figure 1 below shows the structure of the matrices T (left) and B (right) for the violin players network in its undirected configuration for high δ values.

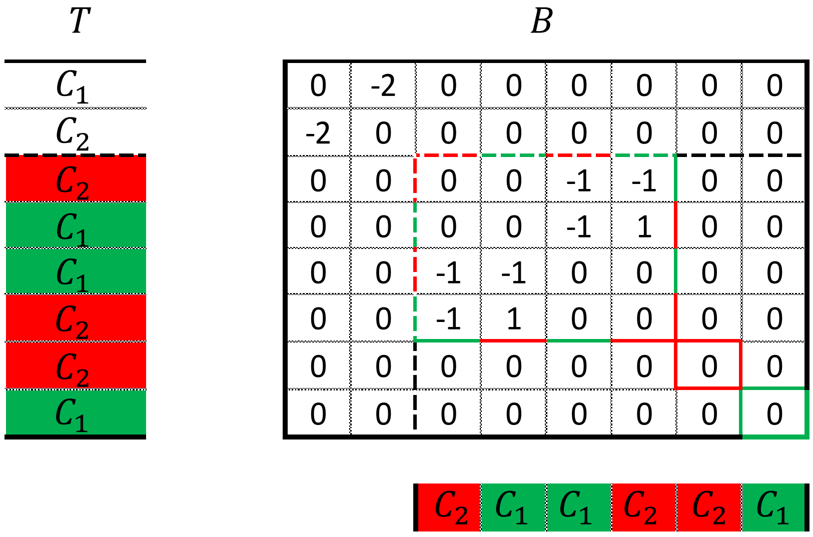

Supplementary Figure 1: Structure of matrix T (left) and complete matrix B (right) for the example of Fig. 6c, in the main paper.

In particular, $B_\perp$ has a block-diagonal structure with three blocks and the left-upper block (with red-green borders) evidences that the two clusters C_1 and C_2 are intertwined.

3.2 Violin players network in its directed configuration 'arrow-head'

We focus on the network shown in Fig. 6 of the main paper, with the non-minimal balanced coloring shown in panel i. Figure 2 below shows the structure of the matrices T (left) and B (right) for the violin players network in its directed configuration with 5 clusters.

T
C_1
C_2
C_3
C_4
C_5
C_4
C_3
C_2

B

0	0	$\sqrt{2}$	0	0	0	0	0
0	0	0	0	0	0	0	0
0	0	0	0	1	0	0	0
0	$\sqrt{2}$	0	0	0	0	0	0
0	0	0	1	0	0	0	0
0	0	0	0	0	0	1	0
0	0	0	0	0	0	0	1
0	0	0	0	0	0	0	0

C_4	C_3	C_2

Supplementary Figure 2: Structure of matrix T (left) and complete matrix B (right) for the example of Fig. 6i, in the main paper. Notice that C_1 and C_5 do not appear in $T_\perp$ because they are trivial.

In this case, $B_\perp$ is block-upper triangular, meaning that some clusters are one-way dependent. In particular, the stability of C_4 depends on that of C_3 (third-last row) and the stability of C_3 depends on that of C_2 (second-last row). The last row (related to cluster C_2) has only zero entries; this implies that the dynamics of each perturbation component η_k depends only on η_k through the matrix Ψ_1 in Eq. (4).

3.3 Two-layer network with interlayer connections oriented in both directions

We focus on the network shown in Fig. 7 of the main paper, with the minimal balanced coloring shown in panel b. We remark that the complete matrices are independent of σ_2.

Fig. 3 below shows the structure of the matrices T (left) and B (right).

$B_\perp$ is a unique 18×18 full block (with red-green borders), which evidences that the two clusters C_1 and C_2 are intertwined.

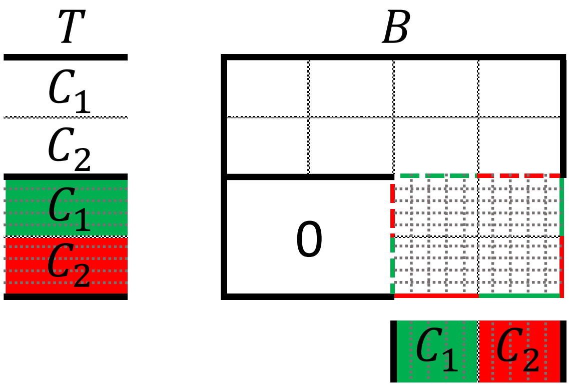

Supplementary Figure 3: Structure of matrices T (left) and B (right) for the example of Fig. 7b in the main paper (minimal balanced coloring). The complete matrices are provided in dataset S2.

Fig. 4 shows the structure of the matrices T (left) and B (right), in the case of non-minimal balanced coloring (main manuscript, Fig. 7e).

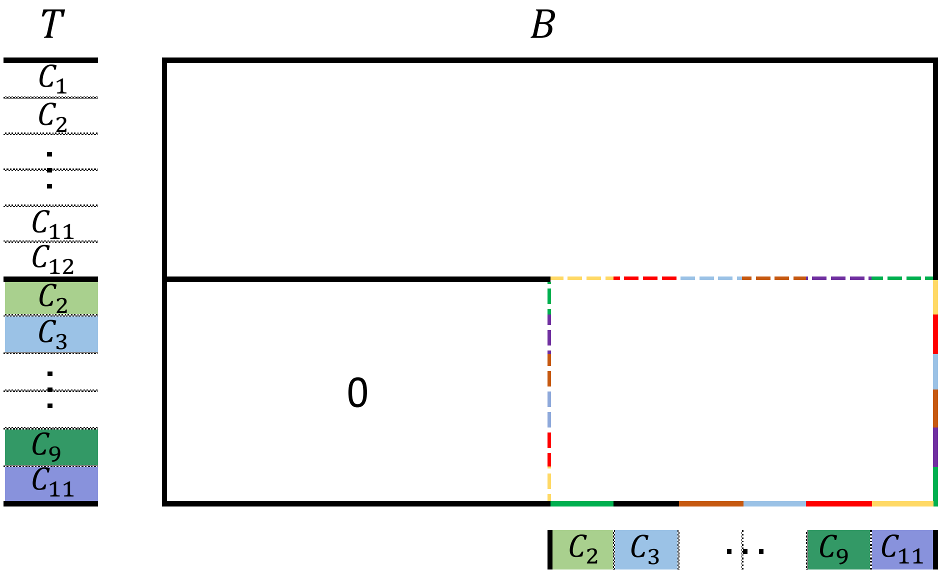

Supplementary Figure 4: Structure of matrices T (left) and B (right) for the example of Fig. 7e in the main paper (non-minimal balanced coloring). The complete matrices are provided in dataset S2.

$B_\perp$ is a unique $(N - Q) \times (N - Q)$ (i.e., 8×8) full block (with multi-color borders), which evidences that the eight non-trivial clusters are intertwined.

3.4 Two-layer network with interlayer connections oriented in one direction

We now consider two networks obtained from the network shown in Fig. 7a of the main paper, by cutting the interlayer connections in one direction. We focus on the case of minimal balanced coloring (Fig. 7b).

First, we keep only the thin red connections from layer I (red) to layer II (blue), removing the thick red connections from layer II to layer I. Fig. 5 shows the structure of the matrices T (left) and B (right).

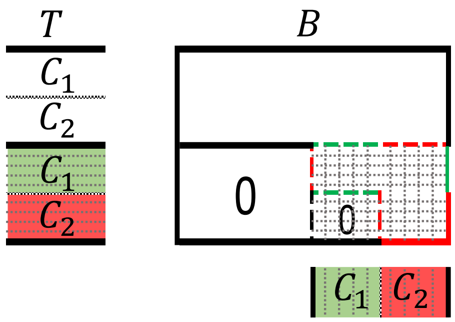

Supplementary Figure 5: Structure of matrices T (left) and B (right) for the network of Fig. 7a in the main paper, removing the connections from layer II to layer I, in the case of minimal balanced coloring (Fig. 7b).

In this case, the 18×18 submatrix $B_{\perp}$ is block-upper triangular and the structure of the corresponding matrix T evidences that cluster C_1 is one-way dependent on C_2.

Conversely, if we keep only the thick red connections from layer II to layer I, removing the thin red connections from layer I to layer II, we obtain matrices with the structure shown in Fig. 6: T (left) and B (right).

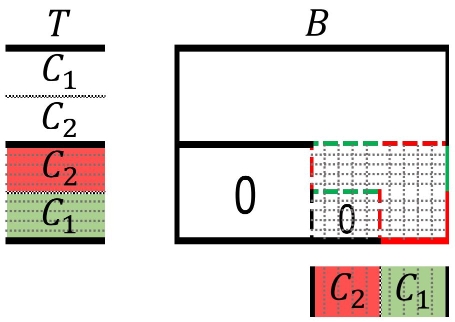

Supplementary Figure 6: Structure of matrices T (left) and B (right) for the network of Fig. 7a in the main paper, removing the connections from layer I to layer II, in the case of minimal balanced coloring (Fig. 7b).

$B_\perp$ is again a unique 18×18 block-upper triangular submatrix and the structure of the corresponding matrix T evidences that cluster C_2 is one-way dependent on C_1.

4 Summary of the proposed method

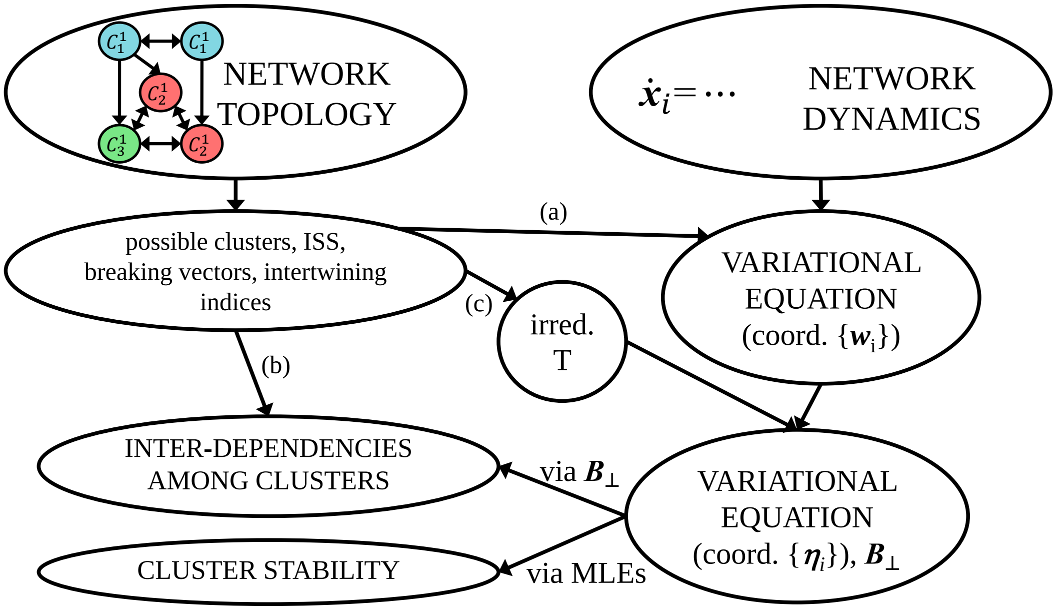

Supplementary Figure 7: Summary of the proposed method. Starting from the network topology, we identify equitable clusters, ISSs, breaking vectors and intertwining indices. Based on these quantities, we can (a) find the Q synchronized motions and the small perturbations $\{\boldsymbol{w}_i\}$ about them, (b) detect interrelations among clusters (easily only for simple networks) and (c) find the irreducible transformation matrix T. Starting from the network equations, we find the equations governing the dynamics of a small perturbation $\boldsymbol{w}(t) = \boldsymbol{x}(t) - \boldsymbol{s}(t)$ about a synchronous solution $\boldsymbol{s}(t)$. Through the matrix T we find (i) the optimal (irreducible) coordinate system $\boldsymbol{\eta}(t)$ to separate the perturbation modes and (ii) the matrix $B_\perp$. The matrix $B_\perp$ evidences the inter-dependencies among clusters (easily also for complex networks) and the variational equations in the coordinate system $\boldsymbol{\eta}$ permits to analyze the stability of each cluster, through the MLEs.

Supplementary Reference

[1] Della Rossa, F. *et al.*, *Nature Communications* **11**, 1–17 (2020).
