# Supplementary material for: One-way dependent clusters and stability of cluster synchronization in directed networks
Source: Nat Commun. 2021 Jul 1;12:4073. doi: 10.1038/s41467-021-24363-7 (PMC8249607; doi:10.1038/s41467-021-24363-7)
Supplement: Supplementary file 2 — Description of Additional Supplementary Files [file 41467_2021_24363_MOESM2_ESM.docx]

Description of Additional Supplementary Files

Title: Supplementary Data 1

Description: Matrices for the example about violin players.

Title: Supplementary Data 2

Description: Matrices for the example about neural activity and chimera states.
